# Tracking behavioral changes of confined sows from the first mating to the third parity

**DOI:** 10.1515/biol-2022-0653

**Published:** 2023-08-04

**Authors:** Fanglu Lv

**Affiliations:** Heilongjiang Polytechnic, No. 5 Xuefu Road, Harbin, China

**Keywords:** stereotypic behavior, confinement duration, parity, sow, activity restriction

## Abstract

The occurrence pattern of stereotypic behavior in high-parity confined sows is still unclear. We continually observed the behavioral changes in activity-restricted sows from the first mating to the end of the third parity. The results showed that the second- or third-parity weaned sows exhibited more vacuum chewing and total oral behavior than the first-parity sows. The vacuum chewing of sows in the second and third pregnancies was significantly more than that of the first-pregnancy sows at each stage of pregnancy, and the total oral behavior changed similarly. The sitting of sows in the second and third pregnancies was significantly more than that of the first-pregnancy sows in the early stage of pregnancy, whereas sitting behavior in the third pregnancy was significantly more than that in the middle stages of the first and second pregnancies. Vacuum chewing or sitting was positively correlated with confinement duration. In summary, vacuum chewing and sitting behaviors in sows confined from the first mating were significantly affected by parity. From the late stage of the first pregnancy to the middle stage of the second pregnancy is the key period of stereotypic behavior development for low-parity sows.

## 1 Introduction

Stereotypic behavior is a type of abnormal behavior with a single performance that is caused by a poor environment and has no obvious function [1,2] and is a chronic stress response [3]. Stereotypic behavior mainly manifests itself as increases in oral behaviors and abnormal postures. There are many tissue lesions [4] and abnormal protein expression in the brains of abnormal sows in movement-limited environments [5].

Although some factors involve body weight [6], sow age [7,8], and the shape or size of farrowing crates [6] can cause stereotypic behavior, a confined environment is a more important factor affecting the stereotypic behavior of sows [9]. On the one hand, oral stereotypies may be caused by food restrictions and confinement [6]. Research shows that food-restricted sows housed in an enriched free pen may not perform vacuum chewing [10,11]. On the other hand, posture changes are mostly caused by physical confinement [12].

Early studies found that confinement can lead to high-parity sows with pupil rigidity [13], vacuum chewing, and other stereotypic behaviors [14,15]. Recent research has shown that short-term space restriction can also cause behavioral changes and that the effects of short-term and long-term restrictions are different [16,17]. For the short-term physical confinement of sows in the lactation stage, stereotypic behavior increased slightly; however, the removal of confinement might have minor short-term positive effects on behavior [18,19,20].

Although there is abundant evidence that a confined environment can lead to the formation of stereotypic behavior, previous studies have only compared sows of different parities [13,21] or sows of different pregnancy stages [17]. These cross-sectional studies showed that confinement duration could affect the stereotypic behavior of sows but could not be used to establish long-term trends in stereotypic behavior. Additionally, there are few studies on the stereotypic behavior of lower-parity sows or the process of behavioral change with confinement time.

Therefore, to explore the occurrence pattern of stereotypic behavior, the present study tracked the behavioral changes in activity-restricted sows from the first mating to the end of the third parity. This longitudinal study will help establish the mechanism of stereotypical behavior and improve management systems to elevate the welfare of sows.

## 2 Materials and methods

### 2.1 Animals and treatment

From April 2016 to July 2017, the experiment was conducted in a grandparent pig breeding farm, Pan Swine Farm, located in Qiqihar, Heilongjiang Province, China. The farm had more than 300 breeding sows and implemented batch farrowing. There was no environmental enrichment for the pigs. Normal ventilation and lighting were used in the houses. During the experiment, the average temperature was 18.0°C (10.2–25.5°C), and the average humidity was 56.2% (35.4–78.9%).

The sows were housed in activity-restricted crates (2.1 m long and 0.6 m wide) with a solid floor throughout their pregnancy. During the lactation stage, sows were housed in farrowing crates (2.15 m long and 1.8 m wide, 0.3 m above the ground). The width of the sows’ stalls was 0.6 m. All crates were made with metal bars so that the sows could see their neighbors. During the weaned stage, each of the four sows were housed in a free pen (3.2 m long and 3.2 m wide) with a solid floor and walls. Four individual feeding stalls were used to prevent fighting. The pregnant sows were fed a gestation diet (before day 84 of gestation) or lactation diet (beginning on day 84 of gestation) at 5:30 and 18:00 daily. The lactating and weaned sows were fed a lactation diet at 5:30, 13:00, and 18:00 daily. All diets were pellet shaped and met the nutrient requirements of swine of National Research Council (NRC) [22] (2012) as shown in Table 1. Water was continuously available from an automatic drinking system. The crates were cleaned daily at 6:00. All management, immune procedures, and disease treatments were are implemented in accordance with the standards of this farm.

**Table 1:** Nutrient composition of the diet of sows (%)

	CP	CA	CF	Ca	TP	NaCl	Lys	ME (MJ/kg)
Pregnant diet	≥15.0	≤12.0	≤12	0.5–1.3	≥0.4	0.3–1.0	≥0.6	≥13.32
Lactation diet	≥16.0	≤10.0	≤9	0.6–1.3	≥0.4	0.3–1.0	≥0.9	≥13.75

The nutrient concentration was calculated from the nutrient composition of the raw materials in the NRC (2012). CP, crude protein; CF, crude fat; CA, crude ash; Ca, calcium; TP, total phosphorus; NaCl, sodium chloride; Lys, lysine.

Fifteen healthy Yorksh**i**re gilts (10 months old, 90–100 kg) of the same batch were housed in the same area of gestation crates from the first mating. The physiological status of each sow was maintained as consistently as possible. They were transferred to the farrowing crates 4 days before the expected parturition day. After 28 days of lactation, sows were transferred to free pens. Subsequently, within 6 days, estrus sows were artificially inseminated and then moved back to gestation crates. Estrus synchronization and timed artificial insemination were used to ensure batch production. Sows were always in the production cycle synchronously until the end of the third parity. Other management practices were conducted following the uniform standards for commercial pig farms. Some sows were culled because of litter size, illness, dystocia, failing to come into estrus within 7 days after weaning or failing to confirm pregnancy. Thus, only six sows were observed throughout the experiment.


**Ethical approval:** The research related to animal use has been complied with all the relevant national regulations and institutional policies for the care and use of animals and has been approved by the Animal Ethics Committee of the Animal Science and Veterinary College of Heilongjiang Bayi Agricultural University.

### 2.2 Behavioral observation

Beginning on the day when the gilts were transferred to the activity-restricted crates, a video recording system was used for 3 consecutive days (if there was something disturbing the sows’ behavior or the video recording, videos from that day were eliminated and recording was extended for 1 day). This observation stage, named stage 0, was used as the baseline. At 1-month intervals, we continuously recorded 7 days during the pregnancy and chose the videos from three of those days for observation by eliminating any disturbed recordings. Behaviors were recorded continuously during the weaning stage. The confinement duration was measured by the 37-day period. All observation stages are shown in Table 2.

**Table 2:** The full name and the corresponding abbreviation of each stage type of the confinement duration

Confinement period	Observing stage	Abbreviation
0	Baseline	
1	Early stage of the first pregnancy	EF
2	Middle stage of the first pregnancy	MF
3	Late stage of the first pregnancy	LF
4	Weaned stage of the first parity	FP
5	Early stage of the second pregnancy	ES
6	Middle stage of the second pregnancy	MS
7	Late stage of the second pregnancy	LS
8	Weaned stage of the second parity	SP
9	Early stage of the third pregnancy	ET
10	Middle stage of the third pregnancy	MT
11	Late stage of the third pregnancy	LT
12	Weaned stage of the third parity	TP

The setting of the behavior parameters in this study referred to the standard described by Zhang et al. [17]. Behavioral categories were classified as oral behavior and posture. The oral behaviors were vacuum-chewing and bar-biting; the postures were lying, standing, and sitting; and the behavioral categories and their corresponding definitions are shown in Table 3. Scan sampling method was used to sample the behaviors at 1 min interval over a 2 h observation period. The duration of each behavior occurring for longer than 5 s was recorded. The total duration of each behavior between 7:00 and 9:00 was recorded with 3 days that we chose above.

**Table 3:** The behavioral categories and the corresponding definitions of this paper

Behavioral categories	Definitions
Bar-biting	Licking, or biting any metal bar of the stall (nosing or rubbing with the stall are elided)
Vacuum chewing	Continuous chewing while no feed is present in the mouth
Lying	Sleep lying and non-sleeping lying
Standing	Sow standing on all fours
Sitting	The forelimbs are upright, and the hindquarters are seated on the floor.

The pre-analysis showed that sows were more active before feeding, but there were few oral behaviors observed. Although the sows were more active after feeding, we chose another peak period of activity for behavioral observation because of the difficulty in distinguishing chewing with or without food in the mouth. The total duration of each behavior between 7:00 and 9:00 was recorded for analysis.

### 2.3 Statistical analysis

SPSS Statistics 20 was used for data analysis. After the data were collected, the normal distribution test and the homogeneity test of variance were performed. A new category of behavior called “total oral behavior” which included bar-biting and vacuum chewing was created for the analysis.

The paired sample *t*-test was used to compare the behaviors of the weaned sows in different parities. As there were different behavioral expressions between sows at different pregnancy stages [17,23], the behaviors of gestating sows in different parities were split into three groups (early stage, middle stage, and late stage) and then analyzed by paired *t*-test. The relationship between confinement duration and behavioral changes was analyzed using a general linear model, and the differences among different stages were analyzed with covariance. The total duration of each behavior is expressed as “mean ± SD”. “*P* < 0.05” was considered significant difference and “*P* < 0.01” was considered extremely significant difference.

## 3 Results

### 3.1 Behaviors of the weaned sows in different parities

**Figure 1:**
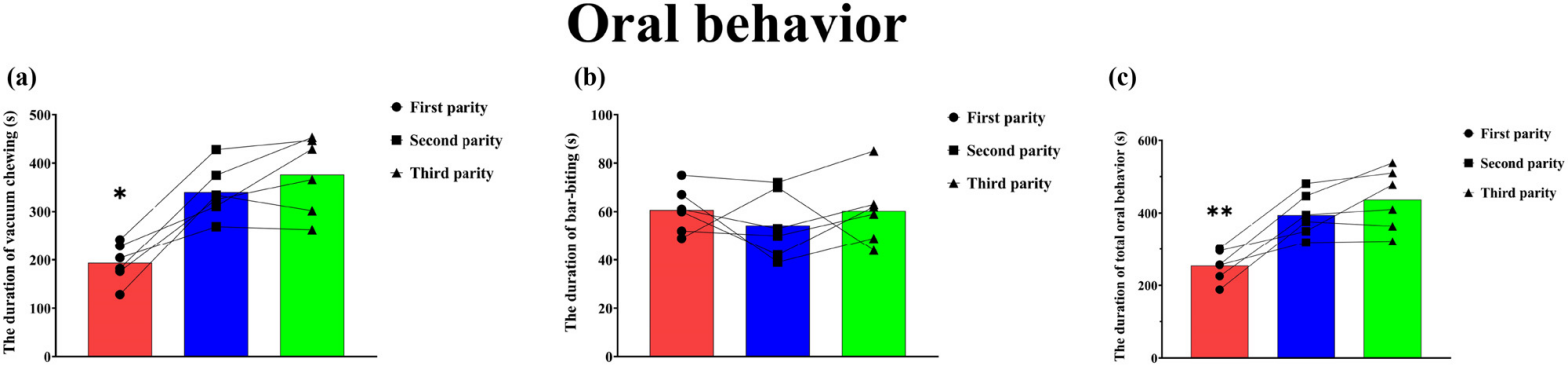
The duration of oral behaviors of the weaned sows. *P < 0.05, **P < 0.01. The lines indicate changes in oral behaviors of each sow at different parities.

As shown in Figure 1a, the vacuum chewing of the second-and-third parity sows (340.33 ± 54.98 and 376.67 ± 80.15, respectively) was significantly more than that of the first-parity sows (193.50 ± 41.11) (*P* < 0.05). There was no significant difference in vacuum chewing between the second- and third-parity sows (*P* > 0.05) nor in the bar-biting behavior of weaned sows in different parities (*P* > 0.05, Figure 1b). The total oral behavior of the second-parity (394.67 ± 60.43) and third-parity sows (394.67 ± 60.43 and 437.00 ± 85.43, respectively) was significantly more than that of the first-parity sows (254.17 ± 43.30) (*P* < 0.01, Figure 1c).

### 3.2 Behaviors of gestating sows in different parities

#### 3.2.1 Behaviors of sows in the early pregnancy stage in different parities

**Figure 2:**
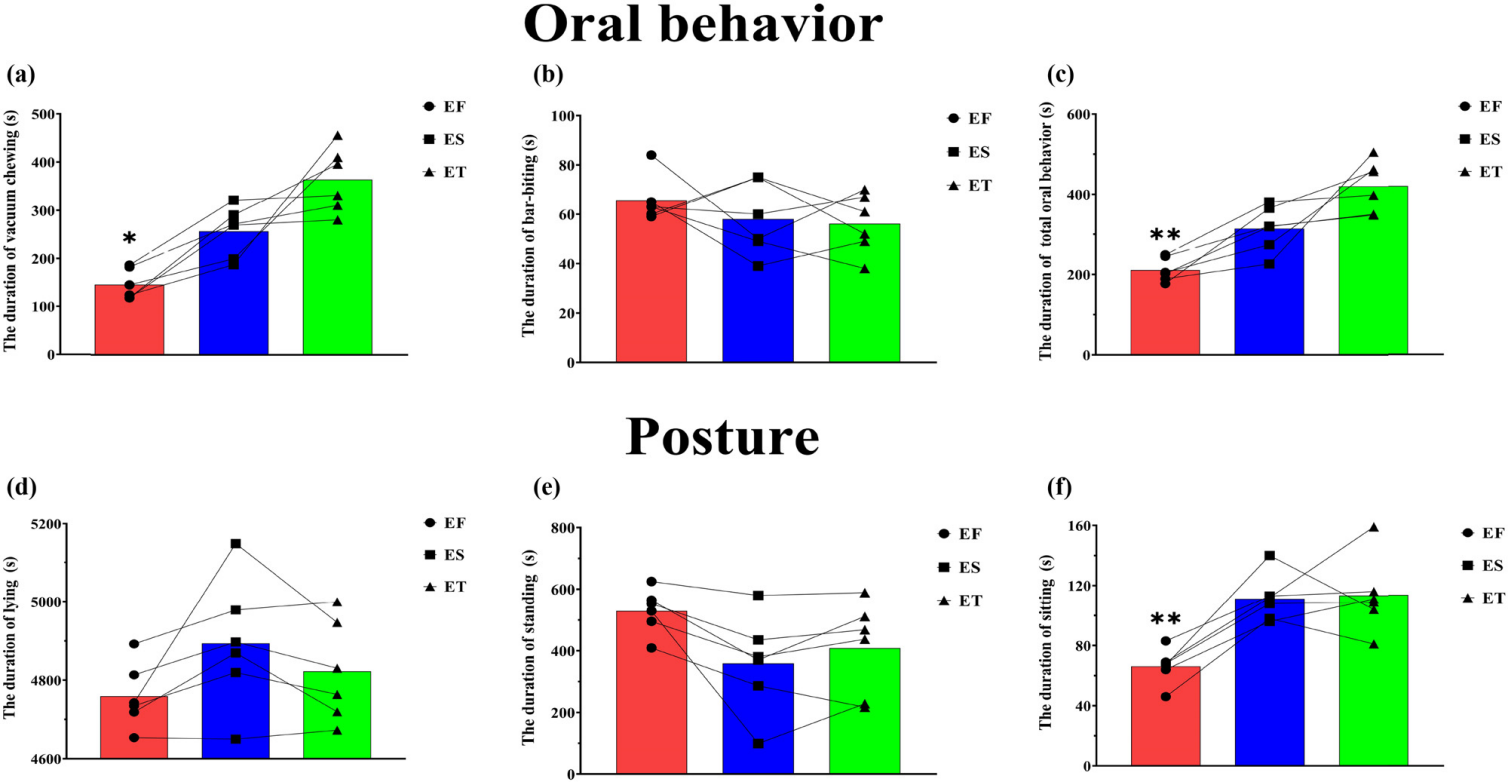
The duration of behaviors of sows in the early pregnancy. *P < 0.05, **P < 0.01. Early stage of first pregnancy (EF), early stage of second pregnancy (ES), and early stage of third pregnancy (ET). The lines indicate changes in behaviors of each sow in the early pregnancy at different parities.

As shown in Figure 2a, sows in the early stage of their second (ES) and third (ET) pregnancies (256.16 ± 21.43 and 363.83 ± 27.49, respectively) exhibited vacuum chewing significantly more than sows in the early stage of their first pregnancy (EF) (134.01 ± 14.5) (*P* < 0.05), and vacuum chewing of the ET sows showed a tendency to be greater than that of the ES sows (*P* = 0.062). There was no significant difference in bar-biting among sows during early pregnancy in different parities (*P* > 0.05, Figure 2b). The total oral behavior of ES and ET sows (314.17 ± 57.44 and 420.00 ± 64.69, respectively) was significantly more than that of EF sows (211.17 ± 29.52) (*P* < 0.01, Figure 2c).

There was no significant difference in lying or standing among sows in early pregnancy in different parities (*P* > 0.05, Figure 2d and e). The sitting of ES and ET sows (111.17 ± 6.45 and 113.33 ± 10.41, respectively) was significantly more than that of EF sows (66.17 ± 4.85) (*P* < 0.05, Figure 2f).

#### 3.2.2 Behaviors of sows in the middle pregnancy stage in different parities

**Figure 3:**
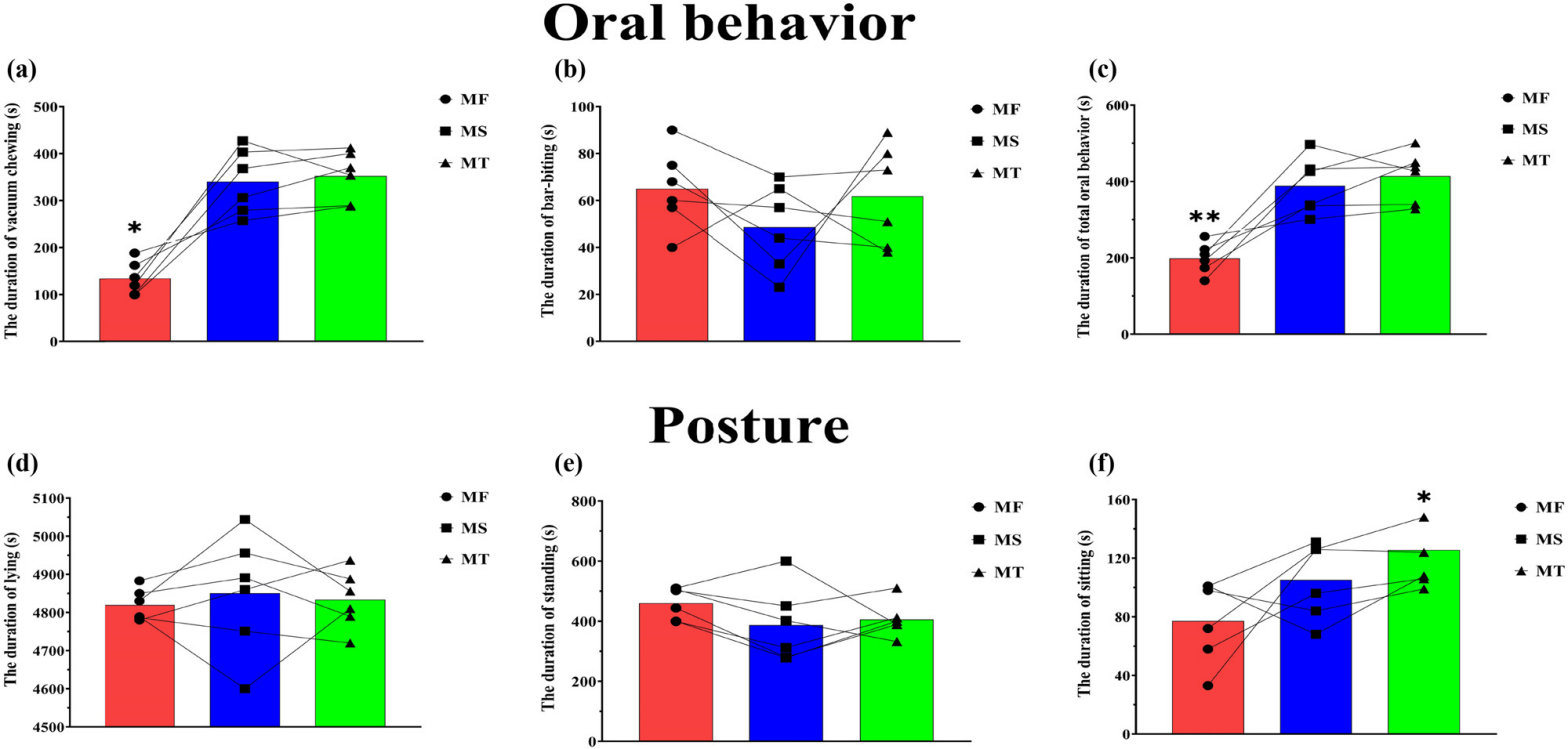
The duration of behaviors of sows in the middle pregnancy. *P < 0.05. Middle stage of first pregnancy (MF), middle stage of second pregnancy (MS), and middle stage of third pregnancy (MT). The lines indicate changes in behaviors of each sow in the middle pregnancy at different parities.

As shown in Figure 3a, the vacuum chewing of sows in the middle stage of their second (MS) and third (MT) pregnancies (340.01 ± 28.33 and 352.17 ± 21.83, respectively) was significantly more than that of sows in the middle stage of their first pregnancy (MF) (134.01 ± 14.5) (*P* < 0.05); there was no significant difference between the MS and MT sows (*P* > 0.05). There was also no significant difference in bar-biting behavior among sows in the middle pregnancy stage in different parities (*P* > 0.05, Figure 3b). The total oral behavior of MS and MT sows (388.67 ± 74.86 and 414.00 ± 67.06, respectively) was significantly more than that of MF sows (199.00 ± 40.05) (*P* < 0.05, Figure 3c). There was no significant difference in postures among sows in the middle pregnancy stage in different parities (*P* > 0.05, Figure 3d–f).

#### 3.2.3 Behaviors of sows in the late pregnancy stage in different parities

**Figure 4:**
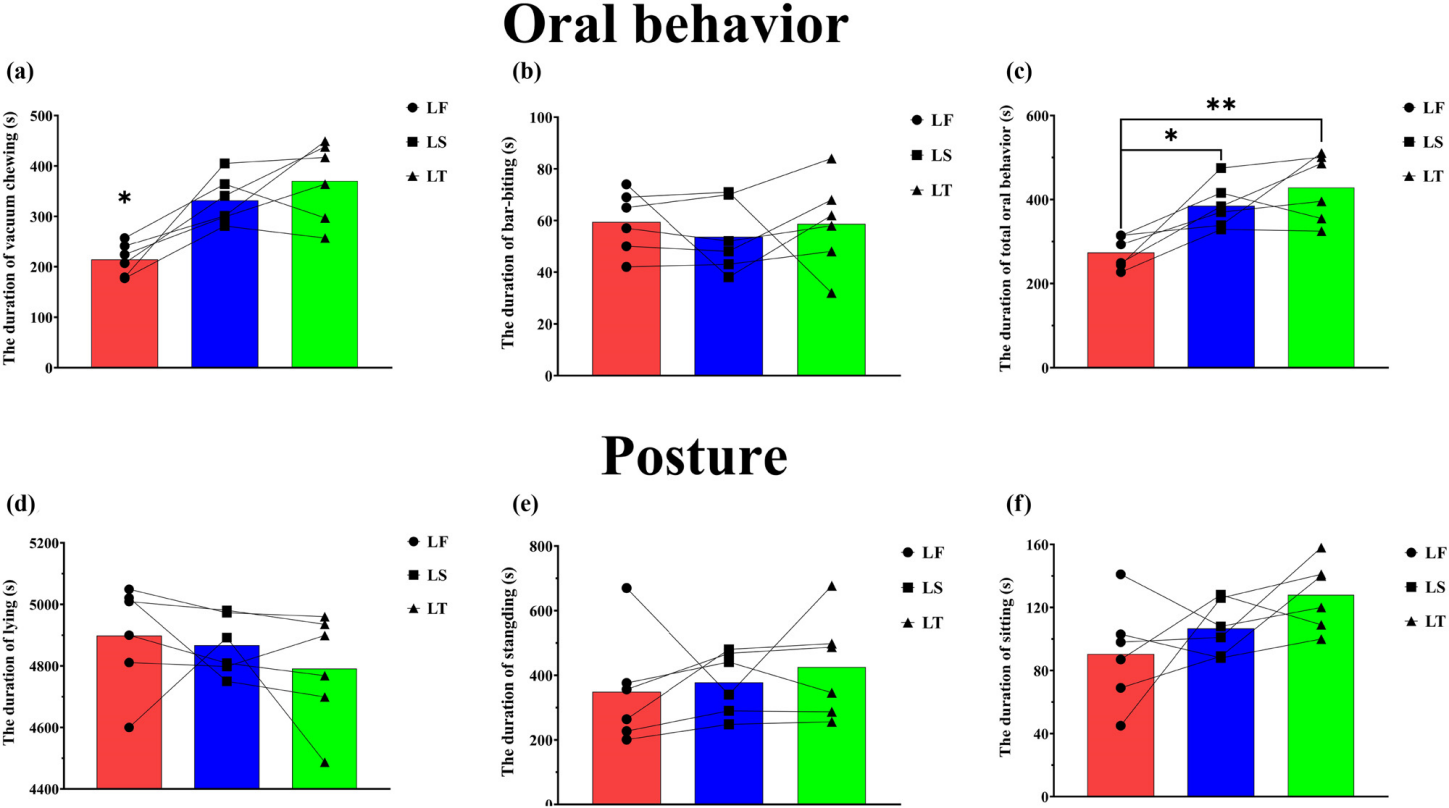
The duration of behaviors of sows in the late pregnancy. *P < 0.05, **P < 0.01. Late stage of first pregnancy (LF), late stage of second pregnancy (LS), and late stage of third pregnancy (LT). The lines indicate changes in behaviors of each sow in the late pregnancy at different parities.

As shown in Figure 4a, the vacuum chewing of sows in the late stage of their second (LS) and third (LT) pregnancies (331.83 ± 19.22 and 370.33 ± 32.25, respectively) was significantly more than that of sows in the late stage of their first pregnancy (LF) (214.17 ± 13.31) (*P* < 0.05). There was no significant difference in bar-biting among sows in late pregnancy in different parities (*P* > 0.05, Figure 4b). As shown in Figure 4c, the total oral behavior of LS sows (385.50 ± 53.91) was significantly more than that of LF sows (273.67 ± 38.41) (*P* < 0.05), and the total oral behavior of LT sows (429.00 ± 80.67) was significantly more than that of LF sows (*P* < 0.01). There was no significant difference in posture among sows in late pregnancy in different parities (*P* > 0.05, Figure 4d–f).

## 4 Relationship between confinement duration and behavioral changes

The regression analysis showed that only vacuum chewing and sitting had a strong correlation with the confinement duration (*R* = 0.988, *R* = 0.978, respectively; *P* < 0.01).

**Figure 5:**
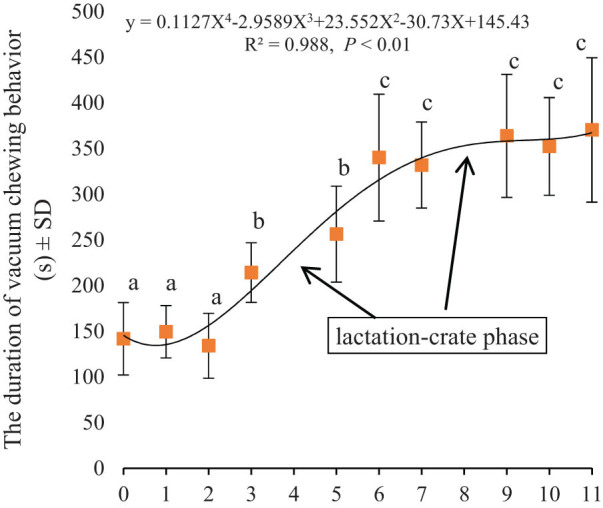
The vacuum-chewing duration time by confinement duration. The lactation period is also included (4, 8). There are significant differences among different stages when marked a, b, and c (*P* < 0.05).

As shown in Figure 5, the regression equation between vacuum chewing and confinement duration was *Y* = 0.1127*X* ^4^ − 2.9589*X* ^3^ + 23.552*X* ^2^ − 30.73*X* + 145.43. The duration of vacuum chewing significantly increased in the LF and MS stages compared with that in the previous stage (*P* < 0.05), and there were no significant changes at other stages (*P* > 0.05).

**Figure 6:**
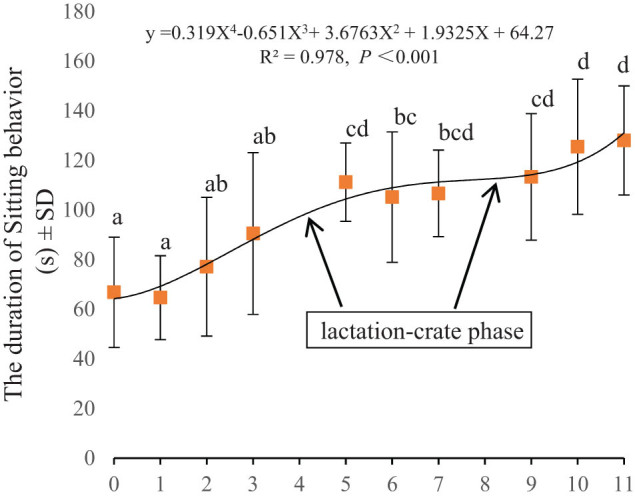
The sitting duration time by confinement duration. The lactation period is also included (4, 8). There are significant differences among different stages when marked a, b, and c (*P* < 0.05).

As shown in Figure 6, the regression equation between sitting and confinement duration was *Y* = 0.319*X* ^4^ − 0.651*X* ^3^ + 3.6763*X* ^2^ + 1.9325*X* + 64.27. The duration of sitting significantly increased in the ES stage (*P* < 0.05) and was significantly longer in the MT and LT stages than in the MS stage (*P* < 0.05). There were no significant changes among the stages of the same pregnancy (*P* > 0.05).

## 5 Discussion

In this study, the vacuum chewing of the second- and third- parity sows was significantly more than that of the first-parity sows at each stage of pregnancy, as well as that in the weaned stage. This is consistent with the effect of parity on vacuum chewing in previous studies [21,24,25,26]. A study by Sekiguchi and Koketsu [27] showed that multiparous sows also had a higher percentage of vacuum chewing than gilts. Thus, the impact of parity on vacuum chewing in low-parity sows was as evident as that on high-parity sows. The reason for the occurrence and increase in vacuum chewing may be the inability to adapt to the high stress caused by confinement. However, there are other possible factors, such as food restriction, feeding methods, and breed [6]. In the present study, the vacuum chewing of sows increased sharply starting with MF sows and changed less drastically from MS to LT sows. Zhang et al. [28] found that the vacuum chewing of gilts increased significantly on the 40th day of pregnancy and then slowed down after the 70th day, which is similar to our results. However, we did not find significant decrease in vacuum chewing after the transfer of sows into the free environment as shown by Zhang et al. [28]. The reason for the conflicting results may be that they provided a more enriched environment for weaned sows. At the same time, their definitions of the pregnancy stages and feeding methods also differ from those of the present study.

Bar-biting might be induced by limited exploratory behavior in a poor environment. Fraser [12] found that bar-biting decreased when straw was provided to sows. However, in the present tracking study, the confinement duration and parity had no significant effect on bar-biting. Some studies showed that there was no difference in bar-biting between the first and second parity, but this behavior increased from the third to the fifth parity [16,28]. In the present study, vacuum chewing was the main form of stereotypical behavior. Although bar-biting of sows did not change significantly, the total oral behavior of the second- and third-parity sows was significantly more than that of the first-parity sows in each stage. Zhang et al. [28] found that the stereotypic behavior of sows increased with parity. Increased vacuum chewing might lead to a maintenance or decrease in bar-biting. Rushen [14] found that tethered sows showed bar-biting and drinking behavior immediately, but vacuum chewing occurred over an extended period of time. Therefore, the mechanism of bar-biting may be different from that of vacuum chewing.

The sitting behavior in the present study increased mainly in the early stage of the second pregnancy, and there was an increasing trend in this behavior during the third pregnancy. Previous studies have found that the sow’s hind limb weight-bearing capacity is negatively correlated with parity [4]. A limited environment leads to reduced movement, increased body fat, and weakened leg muscles, which can increase the frequency of sitting [29]. However, Sekiguchi and Koketsu [27] found no relationship between parity and sitting position. The sitting of low-parity sows suggests that it is caused by additional factors to those above, and the reason may be more complicated. In the present study, the sitting posture was accompanied by vacuum chewing. The sitting of sows might be accompanied by depression, frustration, and other negative psychological reactions [30].

Our results show that lying and standing did not change significantly with parity. However, Zhang et al. [17] found that there was a significant difference in standing and lying among different parity sows without strong regularity. Chapinal et al. [21] also found that standing could be affected by the restriction duration, whereas lying was not affected. Zhang et al. [17] found that the standing of gilts was significantly more than that of high-parity sows on the 25th day of pregnancy. Thus, lying and standing may not be affected by the confinement duration in low-parity sows. These conflicting results may be attributable to the floor, which is important for the posture of the sow [31].

## 6 Conclusions

In summary, vacuum chewing and sitting were significantly affected by the parity of the confined sows. Vacuum chewing was positively correlated with the confinement duration. Sitting also showed a strong correlation with the duration of confinement. Although there are many other factors that may affect stereotypic behavior, we believe that the confinement duration is the leading cause of stereotypic behavior in confined sows, and the period from the late stage of the first pregnancy to the middle stage of the second pregnancy is the key period of stereotypic behavior development in low-parity sows.
